# Machine learning approach for rapid and accurate estimation of optical properties using spatial frequency domain imaging

**DOI:** 10.1117/1.JBO.24.7.071606

**Published:** 2018-12-12

**Authors:** Swapnesh Panigrahi, Sylvain Gioux

**Affiliations:** University of Strasbourg, ICube Laboratory, Strasbourg, France

**Keywords:** machine learning, diffuse optical imaging, spatial frequency domain imaging

## Abstract

Fast estimation of optical properties from reflectance measurements at two spatial frequencies could pave way for real-time, wide-field and quantitative mapping of vital signs of tissues. We present a machine learning-based approach for estimating optical properties in the spatial frequency domain, where a random forest regression algorithm is trained over data obtained from Monte-Carlo photon transport simulations. The algorithm learns the nonlinear mapping between diffuse reflectance at two spatial frequencies, and the absorption and reduced scattering coefficient of the tissue under consideration. Using this method, absorption and reduced scattering properties could be obtained over a 1 megapixel image in 450 ms with errors as low as 0.556% in absorption and 0.126% in reduced scattering.

## 1 Introduction

Concentration of tissue constituents, such as hemoglobin, water, or lipid, provides vital functional information about tissue health, which can assist healthcare practitioners in making important decisions. For instance, quantitative information about tissue oxygenation and blood volume fraction can provide visual assistance during surgery and monitoring tissue condition during and after treatment.^1^ Even though devices that provide this vital information at a single point already exist, there is a constant push and need toward attaining noncontact, real-time, wide-field and quantitative mapping of tissue functional properties. To address this need, spatial frequency domain imaging (SFDI) is a promising candidate within the field of diffuse optical imaging that has already found commercial application in aiding medical treatment.^2^

**Fig. 1:**
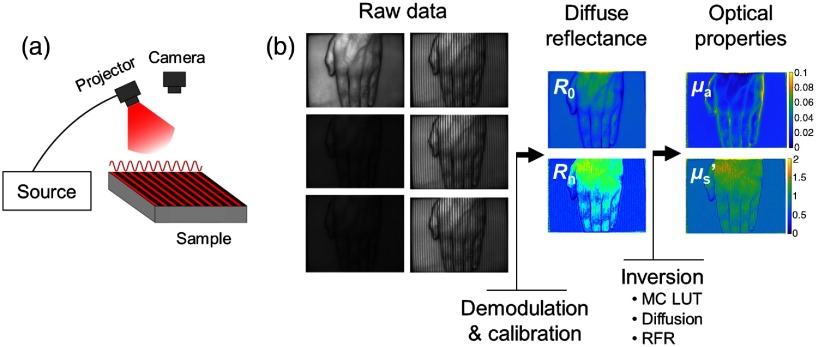
(a) Schematics of a SFDI acquisition system: a laser diode source is fiber-coupled to a digital micromirror device (DMD) projector. Intensity sinewaves are projected and collected using a CCD camera. (b) SFDI processing workflow: raw images are collected at two spatial frequencies and three phases, then demodulated and calibrated to obtain the diffuse reflectance at these spatial frequencies $\left(R_{0},R_{n}\right)$. Finally, an inversion method is used to extract the optical properties maps, namely absorption ($\mu_{a}$) and reduced scattering ($\mu_{s}^{\prime }$).

In essence, SFDI exploits the properties of interaction of spatially modulated light (i.e., structured illumination) with the tissue by measuring the “blurring” of the projected fringes and using light propagation modeling to extract optical properties.^3^ As depicted in Fig. 1(a), a typical imaging system consists of a projection module capable of projecting patterned light at a single wavelength, and a camera for recording images of the field-of-view. Polarizers, arranged in a cross configuration, are commonly used to reduce the effect of specular reflectance and record only diffuse light out of the tissue.

The modulation amplitudes $\left[A_{0},A_{1},\ldots ,A_{n}\right]$ of the sample of interest are obtained at each projected spatial frequency $\left[f_{0},f_{1},\ldots ,f_{n}\right]$ by illuminating, acquiring raw data, and demodulating images using multiple phases^3^ (using SFDI) or a single phase^4^ [using single snapshot of optical properties (SSOP)]. A calibration measurement using a reference phantom with known optical properties is also imaged to obtain modulation amplitudes at the same spatial frequencies $\left[A_{0,\mathrm{ref}},A_{1,\mathrm{ref}},\ldots ,A_{n,\mathrm{ref}}\right]$. The diffuse reflectance $R_{n}$ of the tissue at spatial frequency $f_{n}$ is then given by $R_{n}=\frac{A_{n}}{A_{n,\mathrm{ref}}}*R_{n,\mathrm{ref}}$, where $R_{n,\mathrm{ref}}$ is the simulated diffuse reflectance of the calibration phantom at spatial frequency $f_{n}$. Finally, a light propagation model is used to extract optical properties from the measured diffuse reflectance at several spatial frequencies. This workflow is illustrated in Fig. 1(b).

Because entire images are processed at once in the frequency domain, SFDI has shown the potential to provide very rapid maps of optical properties. The speed of the method can be typically optimized by (1) using a fast acquisition and demodulation method, such as SSOP, and (2) using a fast inversion method to recover the optical properties from the measured data.^5^ It should be noted that both are necessary for SFDI to perform rapidly.

**Fig. 2:**
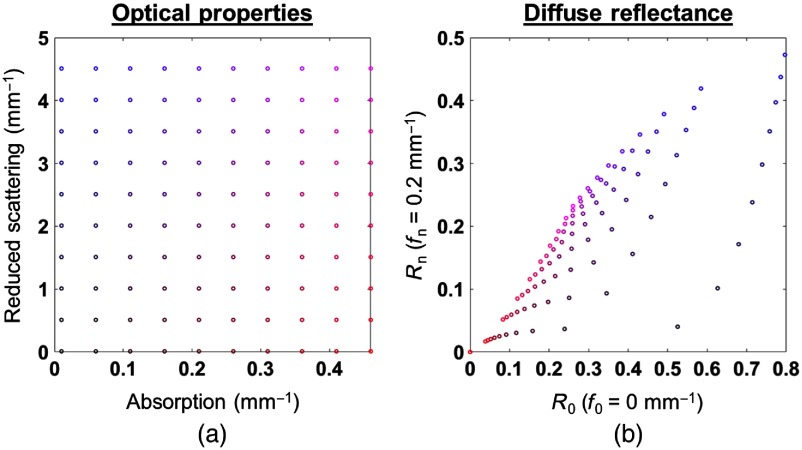
Color-coded mapping between the optical properties and the reflectance $R_{0}$ and $R_{n}$ (corresponding to spatial frequencies $f_{0}=0\;{\mathrm{mm}}^{-1}$ and $f_{n}=0.2\;{\mathrm{mm}}^{-1}$).

To accelerate the entire process, it has been shown that using only two spatial frequencies can provide accurate estimation of optical properties with errors under 7%.^3^^,^^5^^,^^6^ In a two-frequency SFDI scheme, the amplitudes $\left[A_{0},A_{n}\right]$ at low frequency (typically $0\;{\mathrm{mm}}^{-1}$) and a high frequency (e.g., $0.2\;{\mathrm{mm}}^{-1}$) are recorded, and the corresponding reflectance maps $\left[R_{0},R_{n}\right]$ are obtained using the same calibration step. The diffuse reflectance at these two spatial frequencies and the optical properties have a unique, nonlinear, one-to-one mapping relationship.^3^^,^^5^^,^^6^ This nonlinear mapping relationship is shown in Fig. 2 for a wide range of values of the optical properties.

Given the diffuse reflectance images obtained at two spatial frequencies, the task is to solve an inverse problem of obtaining the optical properties, namely the absorption coefficient ($\mu_{a}$) and the reduced scattering coefficient ($\mu_{s}^{\prime }$). The inversion is possible by fitting the diffuse reflectance to either diffusion approximation of photon transport or by using a Monte-Carlo (MC) based simulation of photon migration in tissues.^3^ Using either of these two light propagation models, two basic approaches have been used so far to perform this inversion, namely, least-square fit and look-up table (LUT) method.^3^^,^^5^^,^^6^ On the one hand, precise and robust fitting usually requires diffuse reflectance at multiple frequencies, but it is computationally slow to fit a large number of pixels using either the analytical function provided by the standard diffusion approximation or, especially, with the MC-based simulation. On the other hand, the LUT method uses a pregenerated table of diffuse reflectance for a wide range of optical properties, generally using interpolation to estimate the optical properties.^5^ The LUT method is relatively fast but the computational complexity increases with the density of the grid used to generate the LUT. For a low-resolution LUT, the accuracy of estimation is hampered. A compromise between computational time and accuracy has to be made to use the LUT method. Recent methods that improve the computational time many folds have been proposed.^5^ However, inversion at multiple spatial frequencies and scaling the LUT method to include more parameters other than reflectance has significant computational time and/or memory costs.

In this study, we propose an alternative method for the inversion using a publicly available machine learning technique to estimate the optical properties maps from diffuse reflectance images applied to SFDI. A large set of simulated data using MC was used to train a random forest regression (RFR) algorithm to attain the inversion, allowing in return to obtain directly optical properties maps from diffuse reflectance images in the frequency domain with similar errors than state-of-the-art LUT methods. In the next sections, the method is detailed and its performances evaluated against standard models (diffusion and MC-LUT) on simulated and real experimental data.

## 2 Material and Methods

### 2.1 Random Forest Regressor Method

Machine learning and regression techniques like artificial neural networks and RFR have received an increased interest for processing large amounts of data, and more recently, for replacing time-consuming model-base algorithms in diffuse optics.^7^^–^^10^ What makes such methods attractive is their capacity to perform particularly well in learning nonlinear mappings. In the spatial frequency domain, the mapping between $\left[\mu_{a},\mu_{s}^{\prime }\right]$ and $\left[R_{0},R_{n}\right]$ is strongly nonlinear, as demonstrated in Fig. 2. One possible implementation of machine learning in our case consists of generating a training set with a standard forward model provided by a MC simulation and creating the inverse mapping by a machine learning algorithm.

In this study, RFR, which is an ensemble learning algorithm, has been chosen as it typically works well with the default hyperparameter settings.^10^ The training set was generated by using the MC simulation on MATLAB for a fixed frequency of $0.2\;{\mathrm{mm}}^{-1}$ and refractive index of 1.43. A wide range of optical properties, with $\mu_{a}\in [0,2]\;{\mathrm{mm}}^{-1}$ and $\mu_{s}^{\prime }\in [0.01,15]\;{\mathrm{mm}}^{-1}$, was used and the optical properties space was uniformly and randomly sampled to obtain $10^{6}$ data points. For each pair of randomly generated optical properties, the reflectance $\left[R_{0},R_{n}\right]$ were obtained using the MC simulation. Then, a random forest regressor implemented by scikit-learn package in python^11^ was trained on this dataset to obtain a reverse transformation from $\left[R_{0},R_{n}\right]$ to $\left[\mu_{a},\mu_{s}^{\prime }\right]$. The number of estimator was fixed to 15 for relatively fast calculation and the default minimum samples at leaf were set to 2.^10^ The trained model was saved and used as test over another randomly generated independent test set.

### 2.2 Inversion Methods

Following demodulation and calibration, an inversion method must be used to recover the optical properties [Fig. 1(b)]. Four different methods of inversion were used in this study: the standard diffusion approximation (diffusion), a dense LUT generated from MC simulations of 1000×1000 points in $\mu_{a}$ and $\mu_{s}^{\prime }$ (MC LUT), a low-resolution LUT generated from MC simulation of 100×100 points in $\mu_{a}$ and $\mu_{s}^{\prime }$ (MC LUT low), and the RFR algorithm described previously. To compare computation times, all inversions were performed using MATLAB on a standard PC desktop, have 16 GB of RAM and an Intel I5-7500 processor (6 MB/4T/3,4 GHz).

### 2.3 Imaging System

The instrumental setup was custom built using a digital micromirror device (Vialux, Germany) for the projection of custom patterns, fiber-coupled to a 665-nm laser diode (LDX Optronics, Maryville, Tennessee). The projection system projects a sine wave pattern over a $200\times 150\;{\mathrm{mm}}^{2}$ field-of-view at 45-cm working distance. Images were acquired using a scientific-grade monochrome CCD camera (PCO pixelfly, Kelheim, Germany). Polarizers (PPL05C, Moxtek, Orem, Utah), arranged in a crossed configuration, are used to minimize the contribution from specular reflections at the surface of the sample. A silicone-based optical phantom was used for calibration and built using titanium dioxide (${\mathrm{TiO}}_{2}$) as a scattering agent and India ink as an absorbing agent.^12^ Its large size (210  mm×210  mm×20  mm) accommodates the system’s field-of-view with reduced scattering $\mu_{s}^{\prime }=1.0827\;{\mathrm{mm}}^{-1}$ and absorption $\mu_{a}=0.0117\;{\mathrm{mm}}^{-1}$ at 665 nm.

### 2.4 Validation Experiments

#### 2.4.1 Simulation experiments

An independent test set of 10^6^ optical properties was uniformly and randomly generated for a wide range of optical properties, with $\mu_{a}\in [0,2]\;{\mathrm{mm}}^{-1}$ and $\mu_{s}^{\prime }\in [0.01,15]\;{\mathrm{mm}}^{-1}$ and used to compute diffuse reflectance at two spatial frequencies (0 and $0.2\;{\mathrm{mm}}^{-1}$) using MC simulations. Diffuse reflectance values were then inverted using all models (diffusion, MC LUT, MC LUT low and RFR) and compared.

#### 2.4.2 Phantom experiments

Silicone-based optical phantoms were built using titanium dioxide (${\mathrm{TiO}}_{2}$) as a scattering agent and India ink as an absorbing agent. Six small phantoms having various optical properties ranging from 0.0135 to $0.0437\;{\mathrm{mm}}^{-1}$ in absorption, and from 0.982 and $1.326\;{\mathrm{mm}}^{-1}$ in reduced scattering were made to test the RFR method against the state-of-the-art inversion model, the MC LUT.

Acquisitions were performed by our imaging system at two spatial frequencies (0 and $0.2\;{\mathrm{mm}}^{-1}$) and processed using the RFR and MC LUT methods. A 100×100  pixels region in the neighborhood of a central pixel at each phantom was chosen and mean and standard deviation of the optical properties were computed.

#### 2.4.3 In vivo experiments

Finally, a human hand was measured by our imaging system at two spatial frequencies (0 and $0.2\;{\mathrm{mm}}^{-1}$). Diffuse reflectance values were then inverted using all models (diffusion, dense LUT, low-res LUT, and RFR) and compared.

## 3 Results

### 3.1 Simulation Experiments

**Fig. 3:**
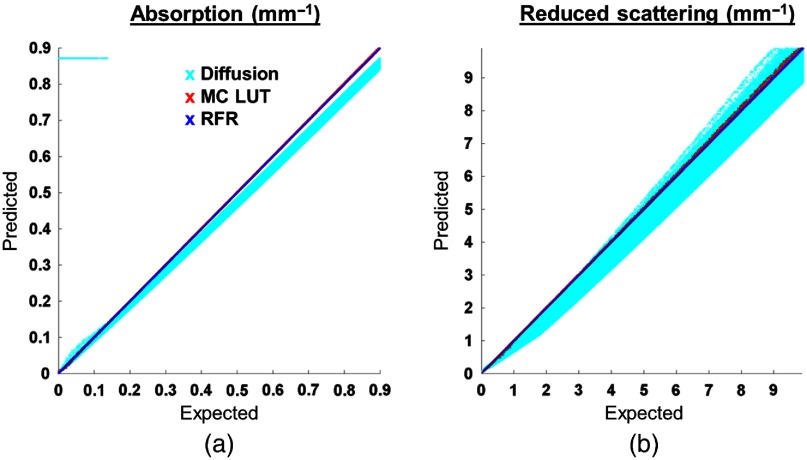
Comparison of inversion methods on randomly generated test set of optical properties and reflectance. The symbols in cyan represent the optical properties predicted by the diffusion approximation, red is the prediction by MC-based LUT, and blue symbols represents the RFR method predictions.

Results from simulation experiments are shown in Fig. 3. The plot shows expected optical properties versus optical properties predicted by the diffusion approximation, MC LUT, and the RFR method, which are shown in cyan, red, and blue, respectively. Overall, the MC LUT inversion shows 0.035% and 0.003% mean relative error in estimation of absorption and reduced scattering coefficients, respectively. However, such large size LUT can take up to 8.8 s to compute the optical properties for a set with $10^{6}$ data points. The RFR method computes the predictions in 0.45 s (nonparallelized) and produces a mean relative of error of 0.556% (in $\mu_{a}$) and 0.126% (in $\mu_{s}^{\prime }$). To obtain, a similar run time on MC LUT, we compare the prediction of the MC LUT Low obtained with 100×100 data points. The run time was improved to 0.43 s, but the accuracy of prediction was deteriorated to 1.86% (in $\mu_{a}$) and 0.097% (in $\mu_{s}^{\prime }$).

### 3.2 Phantom Experiments

**Fig. 4:**
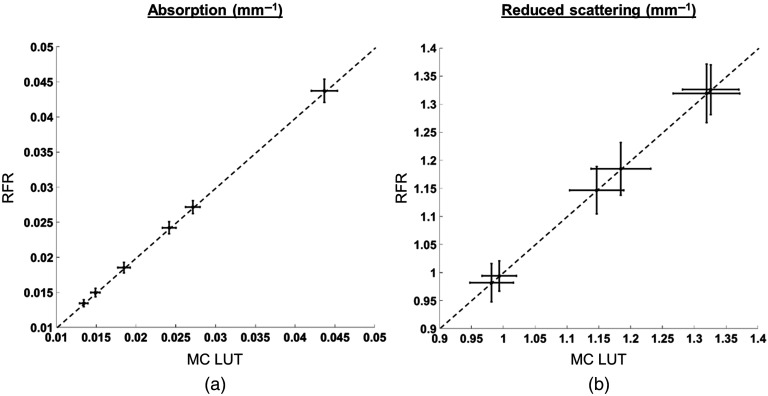
Optical properties of tissue phantoms estimated by the MC-based LUT method (x axis) are compared with the corresponding estimations using RFR method. The values are averaged over 100×100  pixels of a central pixel in each phantom.

Figure 4 shows the comparison of the estimated optical properties on six tissue-simulating phantoms by plotting the mean and standard deviation values obtained from each method against each other. The optical properties estimated from RFR method and the dense LUT method are shown to be similar with a maximum difference of 0.46% in absorption and 0.026% in reduced scattering.

### 3.3 In Vivo Experiments

**Fig. 5:**
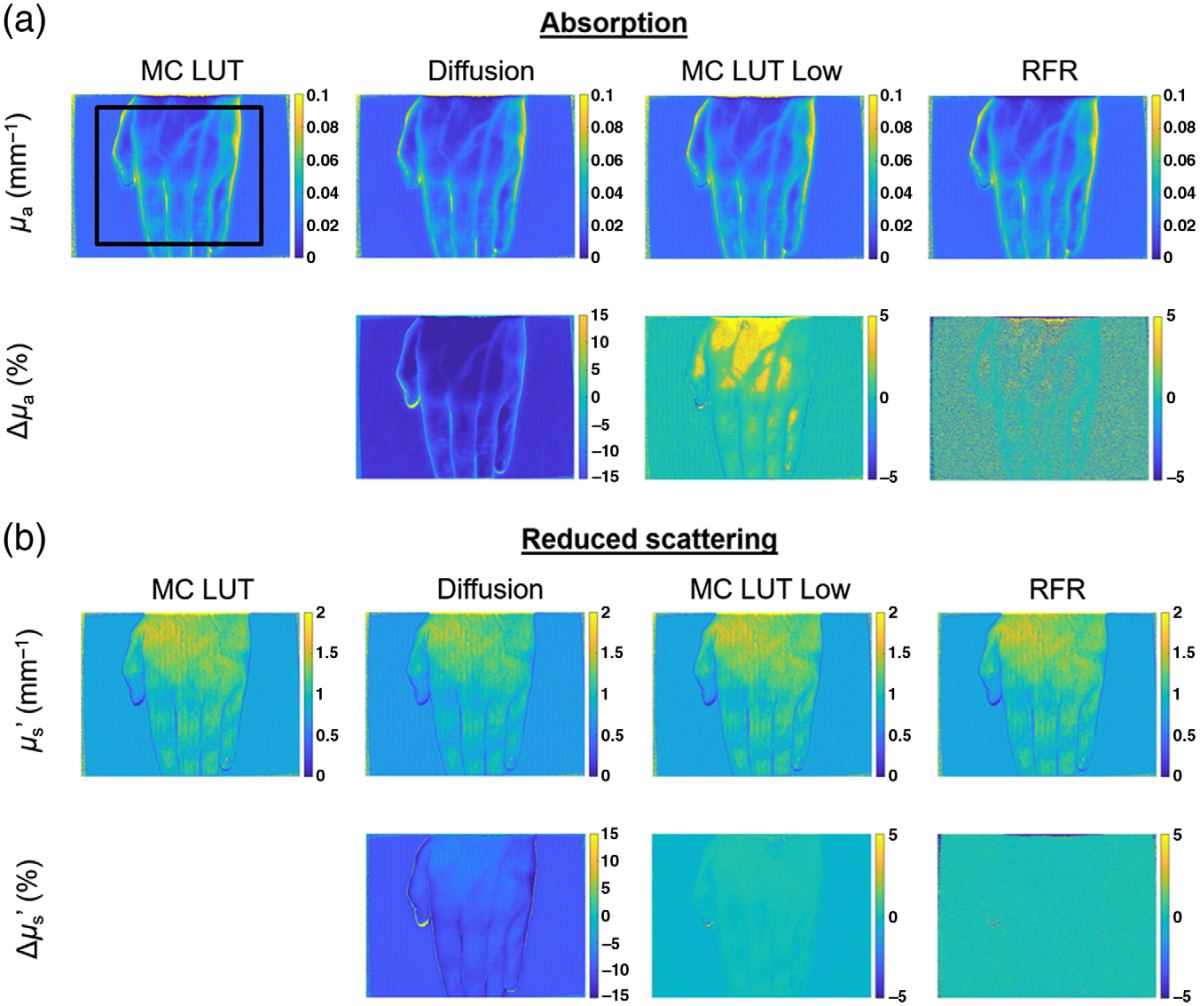
(a) Absorption and (b) reduced scattering results of an *in vivo* hand obtained with all inversion methods (MC LUT, diffusion, MC LUT low, and RFR). For each, the top line shows the optical properties maps and the bottom line shows the percentage error maps in estimation of optical properties compared to the MC-LUT. The black rectangle shows the RoI used for analysis.

The optical properties maps of an *in vivo* human hand obtained from the MC LUT, diffusion approximation, MC MUT low, and RFR methods are shown in Fig. 5. As expected, the MC LUT and the RFR methods perform similarly while the diffusion method visually exhibits deviations in both absorption and scattering. To further quantify these results, the percentage error map with respect to the MC LUT was computed. The diffusion approximation shows a mean percentage error of −16% and −9.2% in estimation of $\mu_{a}$ and $\mu_{s}^{\prime }$, respectively, where the mean is taken over a region indicated by the black rectangle shown in Fig. 5. The mean error in a low resolution MC-LUT is 0.8% and −0.14% in estimation of $\mu_{a}$ and $\mu_{s}^{\prime }$, respectively, while the corresponding error for the RFR method is 0.16% and 0.01% in estimation of $\mu_{a}$ and $\mu_{s}^{\prime }$, respectively.

## 4 Discussion

In this study, we presented a machine learning approach for estimating optical property maps from diffuse reflectance images in the spatial frequency domain. A training set was generated from MC simulations and used to train a RFR algorithm. The RFR method was then tested and validated onto simulations, phantoms, and *in vivo* onto a human hand, against a dense (1000×1000) MC LUT. The accuracy of the RFR method was shown to be slightly degraded compared with the MC LUT in all cases, with differences on average less than 0.16% in absorption and 0.01% in reduced scattering *in vivo*. With very little difference in the accuracy of the recovered optical properties compared to LUTs, the main interest of this work is to propose an alternative approach to estimating optical properties for SFDI without the need of a photon propagation model.

Other machine learning algorithms have been proposed in diffuse optics and in the spatial frequency domain.^7^^–^^10^ The originality of this work is to propose to apply machine learning to process megapixel images rapidly (450 ms) for SFDI while providing accuracies similar to state-of-the-art, dense LUT methods. While our implementation is not faster than previously published work using optimized LUTs,^5^ this study lays the foundation for using machine learning methods for SFDI with the purpose to replace model-based inversion methods to provide images in real-time during surgery. Our machine learning inversion method can certainly be optimized and improved greatly in processing time by using GPU processing, making such approaches potentially as fast as or even faster than LUT-based methods. Coupled with acquisition methods,^4^^,^^13^^,^^14^ both acquisition and processing can be performed in real-time, a requirement to operate in time-constrained environments such as the operating room.

While limited to simple inversion from diffuse reflectance images at two spatial frequencies to obtain optical properties (similar to LUTs), machine learning is particularly interesting for its versatility as it can be easily scaled to more complex data environments for surgical guidance, such as multispatial frequencies analysis, where the instantaneous spatial frequency at each pixel may differ such as during endoscopic SFDI,^15^ multispectral imaging to quantitatively estimate the concentrations of oxyhemoglobin, deoxyhemoglobin, and oxygenation^16^ or fluorescence imaging to quantitatively estimate local the fluorescence concentration.^17^ The method can also be extended to depth-sensitive measurements,^7^ tomography,^18^ 3-D profile correction,^19^ metabolic imaging,^20^ and chromophores mapping^21^ and their combination^22^ in real-time. These measurement modalities make use of a series of models that are usually nonlinear. Given the access to large amounts of simulated or experimental data, these processes can be learned by machine learning models and provide significant improvements in computation time while preserving accuracies of more computationally costly methods. In addition, GPU implementations of machine learning models can help in speeding up the processing of such complex imaging modalities.

## 5 Conclusion

Machine learning methods provide a versatile means for replacing nonlinear models to estimate optical properties from diffuse reflectance images in SFDI without compromising accuracy of estimation. In this study, we presented an RFR algorithm that we trained to perform the nonlinear mapping between diffuse reflectance images and optical properties maps in the spatial frequency domain. The model we presented is capable of computing megapixel maps of optical properties with similar accuracies compared with a standard MC-based LUT. Along with recent developments in real-time acquisition methods in the spatial frequency domain, this work lays the foundation for an integrated imaging system performing acquisition and processing of wide-field images in real-time for surgical applications.
